# Hidden Markov random field models for cell-type assignment of spatially resolved transcriptomics

**DOI:** 10.1093/bioinformatics/btad641

**Published:** 2023-11-07

**Authors:** Cheng Zhong, Tian Tian, Zhi Wei

**Affiliations:** Department of Computer Science, Ying Wu College of Computing, New Jersey Institute of Technology, Newark, NJ 07102, United States; Center for Applied Genomics, Children’s Hospital of Philadelphia, Philadelphia, PA 19104, United States; Department of Computer Science, Ying Wu College of Computing, New Jersey Institute of Technology, Newark, NJ 07102, United States

## Abstract

**Motivation:**

The recent development of spatially resolved transcriptomics (SRT) technologies has facilitated research on gene expression in the spatial context. Annotating cell types is one crucial step for downstream analysis. However, many existing algorithms use an unsupervised strategy to assign cell types for SRT data. They first conduct clustering analysis and then aggregate cluster-level expression based on the clustering results. This workflow fails to leverage the marker gene information efficiently. On the other hand, other cell annotation methods designed for single-cell RNA-seq data utilize the cell-type marker genes information but fail to use spatial information in SRT data.

**Results:**

We introduce a statistical spatial transcriptomics cell assignment model, SPAN, to annotate clusters of cells or spots into known types in SRT data with prior knowledge of predefined marker genes and spatial information. The SPAN model annotates cells or spots from SRT data using predefined overexpressed marker genes and combines a mixture model with a hidden Markov random field to model the spatial dependency between neighboring spots. We demonstrate the effectiveness of SPAN against spatial and nonspatial clustering algorithms through extensive simulation and real data experiments.

**Availability and implementation:**

https://github.com/ChengZ352/SPAN.

## 1 Introduction

Spatially resolved transcriptomics (SRT) profiles gene expression at high resolution while tracking tissue locations of cells (Asp *et al.* 2020). These technologies have greatly accelerated biomedical studies. Mainstream SRT technology can be grouped into two main categories. One is the *in situ* hybridization or sequencing-based technologies with single-cell resolution, such as seqFISH (Shah *et al.* 2016), seqFISH+ (Eng *et al.* 2019), and MERFISH (Moffitt *et al.* 2018). These technologies quantify gene expressions at single-cell resolution, but with only tens to hundreds of genes. Another category is spatial barcoding-based sequencing technologies, including SLIDE-seq (Rodriques *et al.* 2019), SLIDE-seq V2 (Stickels *et al.* 2021), and 10× Visium (10×Genomics). These methods measure the whole transcriptome in spots that contain dozens of cells.

Cell type clustering and identification of SRT data provide the spatial distribution of distinct cell types and are critical analytical steps in many biomedical studies. Like single-cell RNA-seq (scRNA-seq) technology, SRT generates highly sparse and over-dispersed discrete count data, which is statistically and computationally challenging. Many statistical and machine learning models have been proposed for the clustering analysis of scRNA-seq data, including (Satija *et al.* 2015, Kiselev *et al.* 2017, Stuart *et al.* 2019, Tian *et al.* 2019, 2021). However, methods designed for scRNA-seq data have a common issue: they ignore spatial information and simply assume cells are independent. The natural dependencies between neighboring cells or spots are very informative if they can be characterized efficiently and will result in better analysis results. Recently, several clustering methods that capture spatial information have been published. Some deep learning-based models, such as spaGCN (Hu *et al.* 2021), STAGATE (Dong and Zhang 2022), DSSC (Lin *et al.* 2022a), and stLearn (Pham *et al.* 2020), explicitly model the spatial dependency via graph neural network (Kipf and Welling 2017), deep constrained clustering (Tian *et al.* 2021), or spatial-aware data normalization. Statistical methods, such as Bayesspace (Zhao *et al.* 2021), have also been proposed. In these methods, a mixture Gaussian distribution combined with a hidden Markov random field (HMRF) is typically utilized to determine the cell type assignments and smooth the clustering labels in adjacent fields with similar transcriptomics. However, all the aforementioned single-cell and SRT analytical methods are unsupervised algorithms, and users need to assign cell types by aggregated cluster-level expression profiles after clustering analysis. Typical workflows conduct differential expression analysis between clusters to manually label cell types according to over-expressed marker genes. These separate steps can lead to suboptimal results, since they simply ignore marker gene information during clustering analysis.

To utilize the prior information of cell-type specific marker genes, CellAssign (Zhang *et al.* 2019a), and SCINA (Zhang *et al.* 2019b) have been proposed. They assume marker genes are over-expressed in the corresponding cell types, and utilize a mixture model with annotated markers to assign clusters of cells into known cell types. However, these two methods are developed for scRNA-seq data, and do not leverage spatial information in the SRT data.

To address these issues, we propose a statistical spatial transcriptomics cell assignment framework (SPAN) that assigns cells or spots into known types in the SRT data with prior knowledge of predefined marker genes and spatial information. The SPAN model combines a mixture model with an HMRF to model spatial dependency between neighboring spots and annotates cells or spots from SRT data using predefined overexpressed marker genes. The discrete counts of SRT data are characterized by the negative binomial (NB) distribution. Other experimental or technical covariates, such as batch or individual information, can also be incorporated into the model. We evaluate SPAN on extensive simulations and real data experiments and show that SPAN outperforms existing SRT and single-cell clustering methods in various analyses.

## 2 Materials and methods

**Figure 1.**
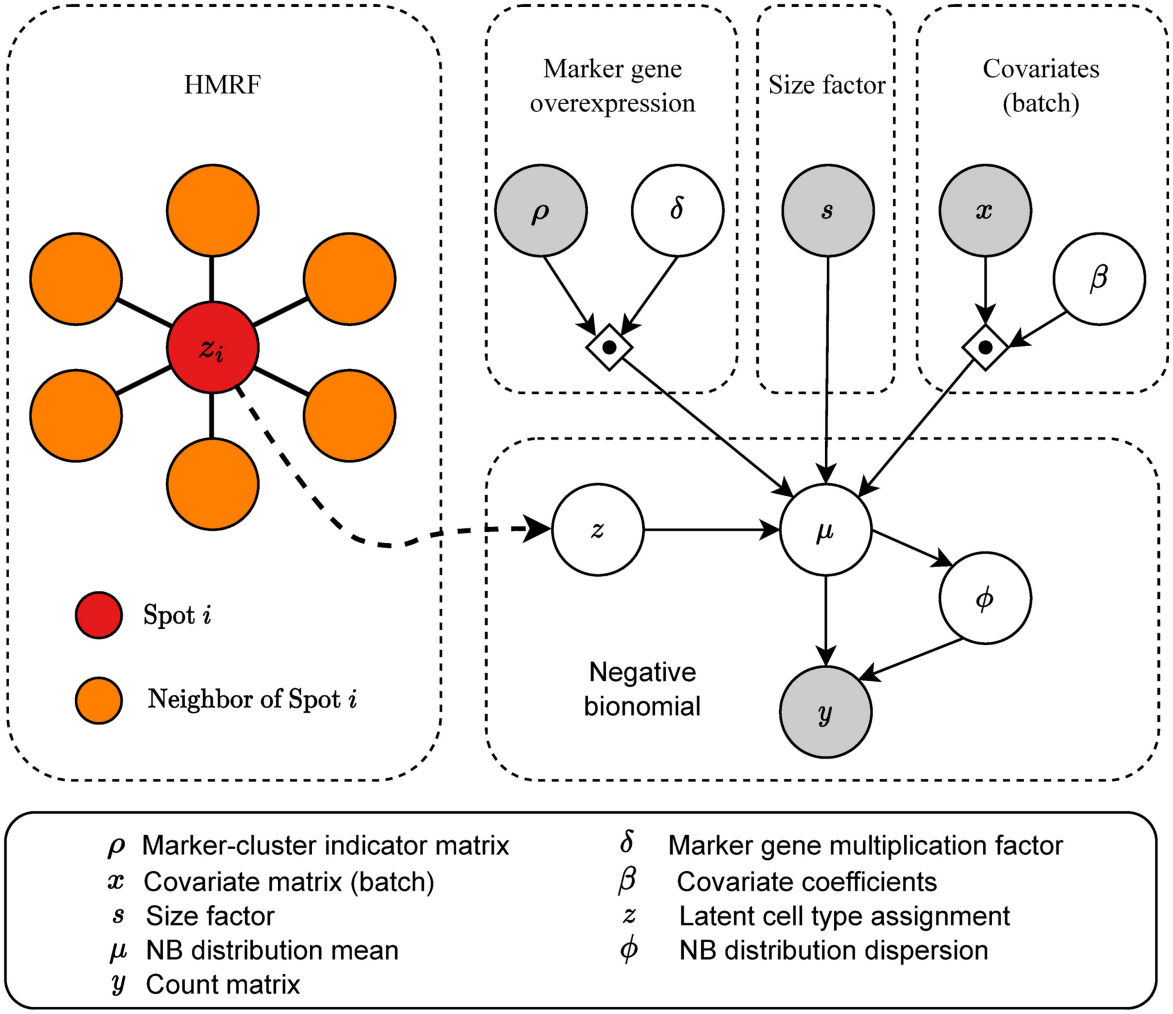
The framework of SPAN. SPAN consists of two modules, a mixture NB distribution modules (right) and an HMRF module (left). SPAN leverages the mixture module to determine region assignments and uses the HMRF module to smooth the clustering results.

The framework of SPAN consists of two modules: a mixture NB distribution module and an HMRF module, as illustrated in Fig. 1. The mixture module takes the gene expression matrix and the marker gene indicator matrix as input to determine region assignments, and the HMRF module uses spatial information to refine the clustering results. The following sections describe the details of these two modules in SPAN.

### 2.1 The NB mixture module

Let ***Y*** represents a spot-by-gene count matrix with *N* spots and *G* marker genes. We assume that these *N* spots can be divided into *K* cluster types. The proposed method models the count matrix ***Y*** by the likelihood of NB distribution. The expression of gene *g* at spot *i* for a latent clustering *k* can be obtained by:


(1) $$\left(y_{ig}|z_{i}=k)∼NB(\mu_{\mathrm{igk}},\phi_{\mathrm{igk}}\right)$$

where the NB distribution is parameterized with mean *μ_igk_* and dispersion $\phi_{\mathrm{igk}}$, and $z_{i}\in \{1,\ldots ,K\}$ is the latent variable indicating the cluster that the spot *i* belongs to.

We follow CellAssign (Zhang *et al.* 2019a) to parameterize the log mean value *μ_igk_* as:


(2) $$\text{log}\;\mu_{\mathrm{igk}}=\text{log}\;s_{i}+\delta_{gk}\rho_{gk}+\beta_{g0}+\sum_{p=1}^{P}\beta_{gp}x_{pi}$$

with the constraint that $\delta_{gk}>0$. *s_i_* represents the size factor for spot *i*. ρ is an indicator matrix derived from the prior knowledge, $\rho_{gk}=1$, if gene *g* is highly expressed in cluster type *k*, and $\rho_{gk}=0$, otherwise. The multiplication factor $\delta_{gk}>0$ models the average log fold change for the marker gene *g* highly expressed in the cluster type *k*, and when $\rho_{gk}=1$, the expression of gene *g* is multiplied by a factor of $e^{\delta_{gk}}$. $\beta_{g_{0}}$ is the base gene expression of gene *g*. ***X*** represents an optional covariate matrix, including the batch and other individual information, and *P* is the number of covariates. *δ_gk_*, $\beta_{g0}$ and *β_gp_* are the parameters that will be learned from the model.

We also place a hierarchical prior on the multiplication factor *δ*, $\delta_{gk}∼\;\text{log}-\text{normal}(\bar{\delta },\sigma^{2})$, where the parameter mean $\bar{\delta }$ and variance $\sigma^{2}$ of the log-normal distribution are set to 0 and 1, respectively. We then add another hierarchical prior on the spot-type assignment $\pi_{k}=p(z_{i}=k)$, where $\left(\pi_{1},\ldots ,\pi_{K})∼\text{Dirchlet}(\alpha ,\ldots ,\alpha \right)$, and *π_k_* and *α* are initialized to 1/K and 0.01, respectively.

Moreover, we set $\phi_{\mathrm{igk}}$ as a sum of radial basis functions (RBF) dependent on the mean *μ_igk_* (Eling *et al.* 2018).


$$\phi_{\mathrm{igk}}=\sum_{j=1}^{B}a_{j}\times \text{exp}(-b_{j}\times {\left(\mu_{\mathrm{igk}}-x_{j}\right)}^{2})$$

where, *a_j_* and *b_j_* are the parameters of RBF, and *B* is the total number of centers of RBF and *x_j_* is the center *j*. The centers are set to be equally spaced apart from 0 to the maximum number of counts *y_ig_*.

Let θ={δ,β,a,b,π} denotes the model learning parameters. The joint distribution of *z* and *y* parameterized by θ is defined by $p(z_{i}=k,y_{i}|\theta )=\pi_{k}\prod_{g}NB(y_{ig}|\mu_{\mathrm{igk}},\phi_{\mathrm{igk}})$. The parameters can be optimized by the Expectation Maximization (EM) algorithm. In E-step, the posterior probability *γ_ik_* can be calculated by:


(3) $$\gamma_{ik}=p(z_{i}=k|y_{i},\theta )=\frac{\pi_{k}\prod_{g}NB(\mu_{\mathrm{igk}},\phi_{\mathrm{igk}})}{\sum_{l}\pi_{l}\prod_{g}NB(\mu_{\mathrm{igl}},\phi_{\mathrm{igl}})}$$

In M-step, θ can be derived by maximizing the *Q* function:


(4) $$Q(\theta )=\sum_{i=1}^{N}\sum_{k=1}^{K}\gamma_{ik}\;\text{log}(p(z_{i}=k,y_{i}|\theta ))$$

Because there is no closed form solution, we can optimize the *Q* function via the gradient descent.

### 2.2 The HMRF module

In SRT data, expression patterns are often correlated in adjacent positions, and two nearby locations tend to have similar clustering assignments. Thus, we apply HMRF to integrate the spatial information and smooth the clustering results.

Let $z=\{z_{1},z_{2},\ldots ,z_{N}\}$ represent the latent spot type assignment. The dependency of *z* can be modeled by a HMRF parameterized with Φ={η,ζ} (Besag 1986). To be more specific, the joint probability of ***z*** is assumed to be


(5) $$p(z;\Phi )\propto \;\text{exp}\;\left(\sum_{1\leq k\leq K}\eta_{k}n_{k}-\underset{1\leq k<l\leq K}{\sum \sum }\zeta_{kl}n_{kl}\right)$$

with the constraint that $\zeta_{kl}>0$, where *n_k_* denotes the number of spots belonging to cluster *k* and $\sum_{k=1}^{K}n_{k}=N$. *n_kl_* is the number of neighbor spot pairs with different group assignments (*k*, *l*). The constraint $\zeta_{kl}>0$ punishes the two neighbor spots having different clustering types.

For a specific spot *i*, the conditional probability of the spot having type *z_i_* = *k*, given the type of all its neighbors is


(6) $$p_{i}(k|\cdot )\propto \;\text{exp}\;\left(\eta_{k}-\sum_{l\neq k}\zeta_{kl}u_{i}(l)\right)$$

where $u_{i}(l)$ represents the number of neighbors of spot *i* having clustering type *l*, and $\zeta_{kl}\equiv \zeta_{lk}$.

Then, the parameters Φ can be estimated by maximizing the conditional likelihood


(7) $$\begin{array}{c} L_{1}(z;\Phi )=\prod_{i=1}^{N}p(z_{i}|z_{\partial i};\Phi )\\ =\prod_{i=1}^{N}\frac{\;\text{exp}\;\left(\eta_{k}-\sum_{l\neq k}\zeta_{kl}u_{i}(l)\right)}{\sum_{k\prime =1}^{K}\;\text{exp}\;\left(\eta_{k\prime }-\sum_{l\neq k\prime }\zeta_{k\prime l}u_{i}(l)\right)} \end{array}$$

where $z_{\partial i}$ denotes the neighbors of spot *i*.

### 2.3 SPAN model

SPAN model is formed by integrating the two modules. We introduce the HMRF as the prior on the mixture NB model to build the SPAN model.

Let Γ={δ,β,a,b}, the conditional probability given clustering assignment *z_i_* is


(8) $$p(y_{i}|z_{i};\Gamma )=\prod_{k}{\left[\prod_{g}NB(y_{ig}|\mu_{\mathrm{igk}},\phi_{\mathrm{igk}})\right]}^{I(z_{i}=k)}$$

where *I* is the indicator function.

The log-likelihood of parameter Γ can be written as


(9) $$L_{2}(y|z;\Gamma )=\sum_{i=1}^{N}\;\text{log}\;p(y_{i}|z_{i};\Gamma )$$

We estimate the model parameters and infer the clustering assignment $z^{*}$, simultaneously. We apply an iterative training process based on iterated conditional models (ICM) (Besag 1986) to estimate Γ and Φ. The model training process is illustrated in Algorithm 1. We first pretrain the mixture NB model to initialize the clustering assignment $z^{\left(0\right)}$. Then, we iteratively compute the parameters Φ and Γ and update the clustering assignment ***z***.

Algorithm 1. SPAN model training process

**Input:** gene expression matirx ***Y***; covariate matrix ***X*** (optional); marker gene—clustering type indicator matrix ρ; neighbor relation ***D***

**Ouput:** an clustering assignment vector ***z***

1: Initialize Γ and pretrain the mixture NB model;

2: Initialize Φ and set $z_{i}^{\left(0\right)}=\text{arg}{\text{max}}_{k}(\gamma_{ik})$;

3: *t *=* *0;

4: **while** (the delta clustering labels >0.1%) and (t≤ the max number of iterations) **do**

5:  $\Gamma^{\left(t\right)}={\text{maximize}}_{\Gamma }L_{2}(y|z;\Gamma )$;

6:  $\Phi^{\left(t\right)}={\text{maximize}}_{\Phi }L_{1}(z;\Phi )$;

7:  **for**i∈{1,2,…,N}**do**

8:   $p(z_{i}^{\left(t+1\right)}|y_{i},z_{\partial i}^{\left(t\right)})\propto p(y_{i}|z_{i}^{\left(t+1\right)};\Gamma^{\left(t\right)})\times p(z_{i}^{\left(t+1\right)}|z_{\partial_{i}}^{\left(t\right)};\Phi^{\left(t\right)})$;

9:   $z_{i}^{\left(t+1\right)}=\text{arg}{\text{max}}_{k}p(z_{i}^{\left(t+1\right)}=k|y_{i},z_{\partial i}^{\left(t\right)})$;

10:  **end for**

11:  t=t+1;

12: **end while**

13: **return *z***;

### 2.4 Model implementation

The SPAN model is implemented in Python3 using PyTorch (Adam *et al.* 2017). Adam (Kingma and Ba 2015) optimizer is used to optimize the $L_{1}(z;\Phi )$ and $L_{2}(y|z;\Gamma )$ and the learning rate is set to 0.01. The hyperparameter *B* is set to 10 by default, and our model has stable performance under different values of *B* (Supplementary Note S3 and Supplementary Fig. S8). The model is first pretrained for one epoch without considering the spatial information and then trained to optimize the entire SPAN model. The experiments are conducted on NVIDIA Tesla P100 GPU.

## 3 Simulation study

### 3.1 Simulation setting

To illustrate the effectiveness of our model, which integrates the marker gene and spatial information for cell type annotation, we compare its performance with two different benchmarks. The first benchmarks apply the standard workflows that use unsupervised clustering methods followed by annotation. These approaches are used for scRNA-seq [Seurat (Stuart *et al.* 2019), SC3 (Kiselev *et al.* 2017) and PCA+Kmeans] and SRT data [Bayesspace (Zhao *et al.* 2021), stLearn (Pham *et al.* 2020) for ST/Visium platform and Giotto (Dries *et al.* 2021) for others]. The second benchmark is a marker gene-based cell annotation approach [Cellassign (Zhang *et al.* 2019a)] designed for scRNA-seq data.

Since SPAN and CellAssign only use the raw counts of marker genes as input, we illustrated the performance of other competing methods based on two inputs: (i) marker genes and (ii) selected high-variance genes (HVGs). We first selected the top 2000 genes (1000 genes for the simulated datasets) by using the mean-variance relationship (Kobak and Berens 2019), and performed principal component analysis (PCA) on the marker genes or selected HVGs. The top 50 PCs were used as input (15 PCs for Bayesspace). For datasets with batch effects, PCs were first corrected by Harmony (Korsunsky *et al.* 2019), respecting the batch IDs for Bayesspace.

Following CellAssign, we mapped the unsupervised clustering results to the ground-truth groups. First, we applied analytic Pearson residuals normalization (Lause *et al.* 2021) to correct sequencing depth and stabilize the variance across marker genes in the count data. Next, we calculated the top 50 PCs for the Pearson residuals normalized count. We computed the average PCs for each ground-truth group and the inferred cluster. Then, we assigned the predicted clusters to the group with the highest Spearman correlation coefficients between the mean PCs of the predicted cluster and all ground-truth groups.

We used Accuracy, macro F1 score and Matthews correlation coefficient (MCC) to evaluate the performance of different methods. To generate the macro F1 score value, we first calculated the F1 score for each cluster with the One-vs-Rest strategy and computed the mean of all F1 scores.

### 3.2 SPAN outperforms competing methods in various settings

To evaluate the model performance, we designed several simulations for different biological scenarios. We extracted the spatial information and ground truth spot type assignment from the sample 151 673 in the dorsolateral prefrontal cortex (DLPFC) (Maynard *et al.* 2021) dataset. We then generated the raw count matrix of 3611 spots of 2500 genes from seven groups via the R package Splatter (Zappia *et al.* 2017). Each simulated cluster has the same number of spots as the corresponding ground truth cluster type in the sample 151 673. We determined the marker genes for each group in the simulated dataset by selecting genes with large differential expression (DEFacGroup value generated by the Splatter). Gene *g* was selected as a marker for group *k*, if *DEFacGroup_gk_* > 1.5. We repeated all experiments ten times under the same setting with different random seeds. The detailed simulation settings are summarized in Supplementary Note S1.

#### 3.2.1 Model performance under different signal strengths

**Figure 2.**
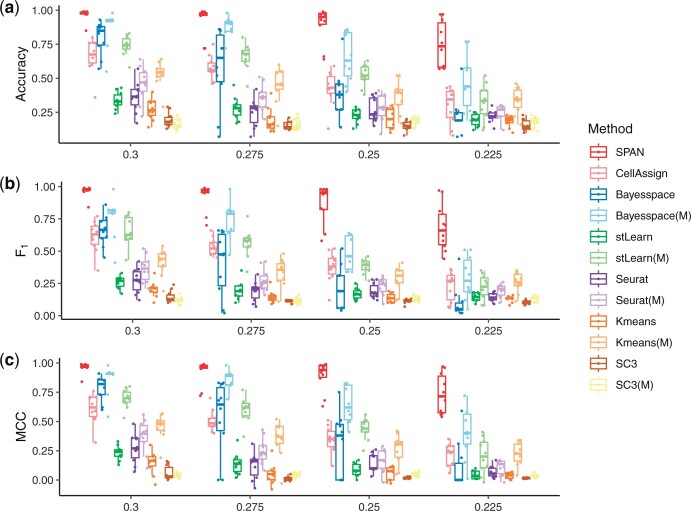
Performance on simulated data with various signal strengths. SPAN and CellAssign use marker genes as input, while other competing methods use HVGs or marker genes (labeled with the letter M in parentheses) as input. (a) Accuracy. (b) F1 score. (c) MCC.

We first evaluated the model performance under different signal strengths. We generated several datasets with different log fold change levels of the gene expression between groups by modifying the variance parameter sigma in the log-normal distribution used by Splatter. Larger variance leads to stronger signal strength and larger distances between different clusters. We varied the sigma from 0.3 to 0.225 and fixed other parameters. The accuracy, F1 score and MCC are illustrated in Fig. 2, and one clustering assignment example is shown in Supplementary Fig. S1. Points in Fig. 2 represent the results on simulation datasets with different random seeds. We demonstrated the performance of SPAN and CellAssign using marker genes as input, and other competing methods using HVGs and marker genes (labeled with the letter M in parentheses) as input, respectively. We note that, for all methods, the performance decreases as the signal strength decreases. Except for two marker gene-based approaches, SPAN and CellAssign, other methods leveraging marker genes as inputs often achieve better performance compared to those utilizing HVGs. This proves that marker genes can provide useful information for determining cluster types, while other nonmarker genes may introduce additional noise information. Moreover, by comparing the marker gene-based approach to the unsupervised methods, we find that SPAN yields better performance than Bayesspace and stLearn, and CellAssign also achieves higher performance than Seurat, SC3 and Kmeans. It indicates that introducing prior knowledge of marker genes can help cluster annotation. Furthermore, the higher accuracy and MCC achieved by the three spatial methods SPAN, Bayesspace and stLearn demonstrate the efficiency of considering the spatial information in determining spot type in SRT. Finally, the cluster assignment examples show that the SPAN, Bayesspace and stLearn can generate smooth clusters by considering spatial information.

#### 3.2.2 Model performance given imperfect marker gene information

We then investigated the performance of our model under different levels of inaccurate marker genes in two scenarios. The first case assumes that the simulated data may contain some cluster-irrelevant genes (nonmarker genes), but other marker genes are assigned to the corresponding groups. The second case assumes that the simulated data does not have nonmarker genes but some markers are assigned to incorrect clusters.

**Figure 3.**
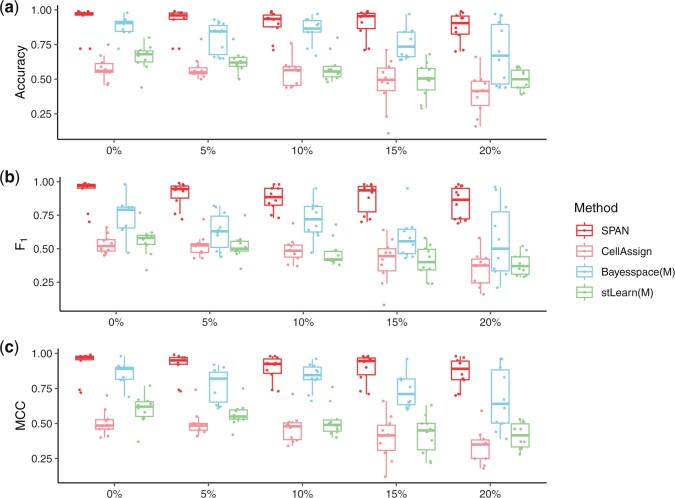
Performance on simulated data with various level of nonmarker genes. All methods use marker genes as input. (a) Accuracy. (b) F1 score. (c) MCC.

In the first case, we randomly replaced some marker genes with other nonmarker genes and randomly assigned a group type to each fake marker gene. We fixed the sigma and varied the ratio of nonmarker genes from 0% to 20%. A ratio equal to 0 means that we do not introduce any fake marker genes, and the larger the ratio, the more fake markers. We compared the performance of marker gene-based approaches, SPAN and CellAssign, and two spatial clustering methods, Bayesspace and stLearn, using only marker genes as input. As shown in Fig. 3, when the ratio increases, the performance of all methods decreases, which indicates that incorrect marker genes can deteriorate the performance. Moreover, compared to SPAN, the performance of other benchmarks drops quickly when the rate increases. In the second case, we varied the ratio of incorrectly assigned markers from 0% to 20%. We compared the performance of SPAN and CellAssign. Similarly, SPAN achieves better performance, as illustrated in Supplementary Fig. S2.

### 3.3 SPAN is robust under different spatial dependencies

In SRT, expression patterns are often correlated, and adjacent locations are more likely to belong to the same group. While we expect this cell type smoothness assumption to hold well in most SRT, there are also cases where this is decidedly not the case and more diverse spatial dependency patterns are observed. Therefore, we conducted the following experiments to see how the performance of SPAN persists under different spatial dependencies.

**Figure 4.**
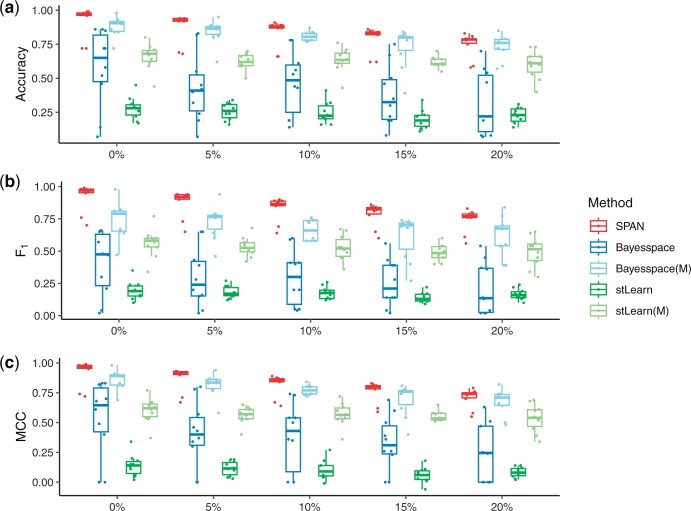
Performance on simulated data with various spatial noise. SPAN uses marker genes as input, while other competing methods use HVGs or marker genes (labeled with the letter M in parentheses) as input. (a) Accuracy. (b) F1 score. (c) MCC.

Firstly, we introduced spatial noise by randomly switching the gene expression and related spot type assignments of spots between different groups. We fixed the sigma and varied the switch ratio from 0% to 20%. A ratio equal to 0 means that we do not switch the spot position, and the larger the ratio, the greater the signal noise, and the less smooth the group assignment. We tested the performance of three spatial clustering methods, SPAN, Bayesspace and stLearn, under different levels of spatial noise. Figure 4 illustrates the performance under accuracy, F1 score and MCC, and Supplementary Fig. S3 shows a cluster assignment example. We can see that as the ratio increases, the performance of all algorithms decreases. Since spatial clustering methods assume that closer spots should have similar assignments, the higher the spatial noise, the worse the performance. However, we expect that the model can still distinguish regions based on the marker gene information, even in the presence of some spatial noise. SPAN yields the best performance under all these settings, which demonstrates the contribution of introducing prior information of cell type corresponding marker genes.

Secondly, to further demonstrate the performance of the proposed method when the smoothness is not strong, we directly simulate a scenario where different cell types are mixed. Specifically, spatial information is extracted from a Slide-seq cerebellum dataset from the RCTD (Cable *et al.* 2022), which is then used for data simulation (Supplementary Fig. S4d). These simulated datasets have 4122 cells from seven distinct cell types (Supplementary Note S1). Only the cell type 3 clearly exhibits strong spatial correlation, while other clusters, such as 1 and 7, are intermixed. We find that when the signal strength between cell types is strong enough (sigma = 0.3), the gain from modeling spatial dependency remains positive, despite the imperfect smoothness. As a result, our methods can outperform other benchmarks (Supplementary Fig. S4). However, when the signal strength between cell types is weak, our model may not benefit significantly from modeling the weak type dependency between neighboring cells, leading to only comparable performance.

Thirdly, we also evaluated the model performance using the spatial information extracted from another ST platform. This melanoma dataset (Thrane *et al.* 2018) contains 293 spots from 4 groups. It is noted that some cell types contain only a limited number of spots, which implies a small sample size and presents a challenging case when marker gene information connot be used. Similarly, we observed that our proposed method yielded better performance than the competing methods under different signal strength (Supplementary Fig. S5), thanks to its integration of marker gene information.

### 3.4 SPAN still prevails when imperfect prior information is provided

The proposed method relies on prior knowledge of the number of cell types, *K*, and the marker-cell type indicator matrix, *ρ*. It is common that our prior knowledge about cell types may not be perfect, with one cell type not provided (corresponding to *K* − 1), or additional cell types provided but not existing in the given sample. It is interesting to test whether SPAN can assign the cells with missing cell type information to NAs (“to be determined”), and if SPAN can decline to assign any cells to additional cell types when they are provided but not actually present in the given sample. Thus, we next evaluated the model’s robustness with imperfect prior cell type information that reflects real-world scenarios (Supplementary Note S1). Specifically, for the given seven cell types (Layers 1–7), we removed the marker gene information of one layer (Layer 1, marked as NA) while adding marker gene information of four nonexistent cell types (Layers 8–11). Not surprisingly, SPAN gave an almost perfect assignment for the cells with correct marker gene information (Layers 2–7, Supplementary Fig. S6d). Interestingly, SPAN could successfully assign some cells from Layer 1 with missing marker gene information to NAs. However, it might also misassign some cells in Layer 1 to other layers, including the nonexistent cell types. Overall, our model still outperformed Cellassign (Supplementary Fig. S6a–c), despite the imperfect prior information.

### 3.5 SPAN effectively accounts for batch information

We demonstrated the performance of SPAN and Bayesspace for batch correction. Similar to the previous simulation, we extracted the spatial information and spot clusters from the samples 151 673 and 151 674 in the DLPFC dataset, and generated 3611 and 3635 spots for two batches via Splatter, respectively. For batch correction, SPAN takes batch IDs as inputs, while Bayesspace cannot handle batch effects. Hence, we applied Harmony (Korsunsky *et al.* 2019) to correct batch effects, respecting the batch IDs, and the Harmony-corrected PCs were used for Bayesspace analysis. As illustrated in Supplementary Fig. S7, SPAN also outperforms Bayesspace.

## 4 Application to real data

**Figure 5.**
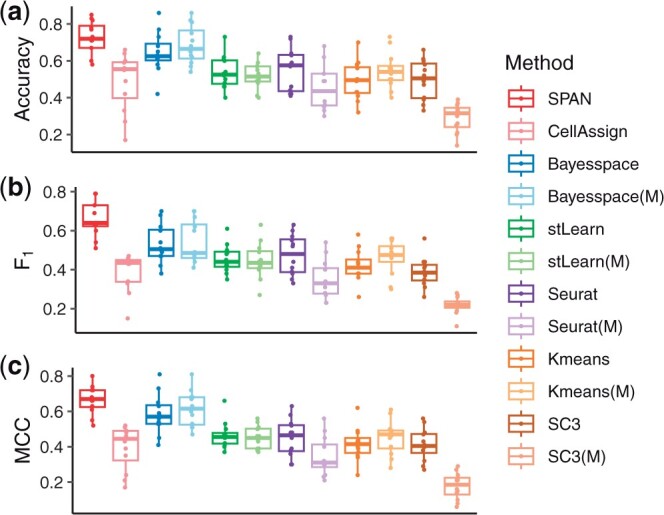
Performance on DLPFC dataset. SPAN and CellAssign use marker genes as input, while other competing methods use HVGs or marker genes (labeled with the letter M in parentheses) as input. (a) Accuracy. (b) F1 score. (c) MCC.

We applied SPAN to three real datasets (Supplementary Note S2) to evaluate the model performance. The DLPFC dataset (Maynard *et al.* 2021) has 12 samples, each of which has 3000–4000 spots from five to seven groups. We use a list of 91 genes that were annotated and compiled in previous studies (Molyneaux *et al.* 2007, Zeng *et al.* 2012, Maynard *et al.* 2021, Lin *et al.* 2022a) as the input for SPAN (Supplementary Table S1). Figure 5 illustrates the performance of 12 samples under accuracy, F1 score and MCC. SPAN achieves the best performance compared with other marker gene-based methods and unsupervised clustering methods. Similar to the clustering results observed on the simulated dataset, we note that introducing prior knowledge of marker genes and spatial information can improve the model performance. Supplementary Figure S9 shows the ground truth and predicted cluster assignments generated by different methods of sample 151 569. The assignments generated by our model are more similar to the ground truth.

We also evaluated the performance of SPAN for batch correction on the eight samples with a total of 32 397 spots from seven groups in the DLPFC dataset (Supplementary Fig. S10). We assumed that the eight samples were from different batches. SPAN takes the batch IDs as inputs, while PCs corrected by Harmony are used for Bayesspace clustering. Supplementary Figure S10a–c show the overall accuracy, F1 score and MCC for the entire eight batches (samples). Supplementary Figure S10d–f illustrate the performance for each batch (sample) individually. SPAN achieves better performance compared to Bayesspace. It shows that SPAN can handle the batch effect by incorporating it as a model covariate. Furthermore, we compare the performance of individually training the eight samples and training them jointly with the batch information as inputs. As shown in Supplementary Fig. S11, SPAN achieves similar performance under these two settings. It proves the model’s ability to adjust the batch effect.

For the other two real datasets, we generate the marker genes via differential expression analysis using DESeq from the R package DESeq2 (Love *et al.* 2014). A detailed dataset description and selected marker genes are provided in Supplementary Note S2 and Supplementary Table S1, respectively.

The Adult Mouse Brain (FFPE) dataset, which is provided by the 10× scRNA-seq platform (Zheng *et al.* 2017), contains 2264 spots from nine groups. 259 genes are used as input for SPAN. Supplementary Fig. S12 illustrates the model performance, and SPAN again achieves the best performance.

**Figure 6.**
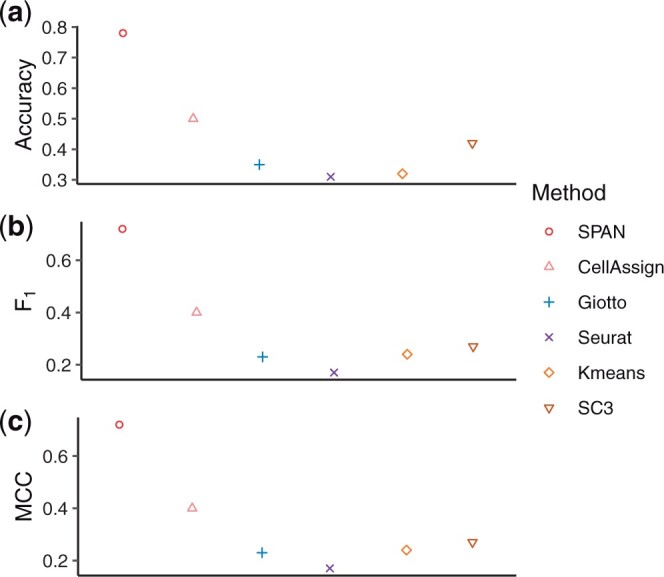
Performance on osmFISH (mouse cortex) dataset. (a) Accuracy. (b) F1 score. (c) MCC.

We then applied SPAN to the osmFISH (mouse cortex) dataset (Codeluppi *et al.* 2018). This dataset contains 4839 cells from 11 groups. Due to the low dimension of features, we use all 33 genes as input for all methods. Unlike the 10× platform which has the apparent neighbor relationship between spots, osmFISH dataset only provides cell coordinates. To generate the cell neighbor relationship, we constructed the neighbor structure by finding the k-nearest neighbors of each cell. In the experiments, we set *k *=* *15. Figure 6 illustrates the model performance, and SPAN outperforms the other competing methods.

Finally, we demonstrated the running time of SPAN by comparing the running time with other competing methods on the 12 DLPFC samples. As shown in Supplementary Fig. S13, SPAN requires less running time than the other two spatial clustering algorithms.

## 5 Discussion

Existing clustering algorithms for SRT data assign cell types by leveraging spatial information in an unsupervised way, while other cell annotation methods fail to use spatial information efficiently. In this article, we propose SPAN to annotate cells or spots in SRT data by integrating the prior knowledge of cell/spot-type marker genes and spatial information. SPAN leverages cluster corresponding marker genes to determine the assignments and applies the HMRF to smooth the results. SPAN can also jointly handle spots from multiple batches (samples) by taking the batch ID as input. We have demonstrated the performance over other unsupervised clustering methods and cell annotation algorithms on different simulation scenarios and real datasets.

This study finds that the prior information of canonical marker genes described in the literature or from curated datasets can improve the accuracy of cell type assignments. Other studies have also demonstrated the utility of marker gene information in clustering cells (Tian *et al.* 2021, Lin *et al.* 2022a,b). Together, these promising results suggest the potential of leveraging marker gene information and emphasize the need for integrating it into the development of computational methods for various analytic tasks in single-cell studies, such as cell–cell communication, trajectory inference, and multiomics data analysis. Moreover, given the incomplete and imperfect cell type information, handling unknown cell types and/or nonexistent cell types remains a challenging problem. Type I error control is not yet considered in cell type assignments. Having type I error control may lead to annotating unknown cell types as NAs, as desired, or assigning multiple cell types to a cell when uncertainty is high. Type I error control for multiple assignments is relevant to conformal prediction, a field that has received much attention in recent years (Xu *et al.* 2023). All of these aspects could be intriguing topics for future research.

Although SPAN achieves good performance for SRT data, there are still some limitations that can be improved. First, we used the k-nearest neighbors approach to determine the neighbors based on the Euclidean distance between cells. However, alternative methods for generating neighbors can be further investigated. Second, in SRT, a spot may contain different types of cells, such that the cell-type markers cannot reflect the entire gene expression of the spot. When two or more types of cells are evenly distributed in a spot, it may greatly impact the model performance. This is likely to occur at the border of two groups. Implementing some downstream analysis may provide more accurate predictions. Third, our model only applies the first-order Markov model to simulate the correlated expression pattern in the adjacent spots in SRT data. However, it is possible to explore more complex spatial patterns by incorporating second or higher-order neighbors. Thus, we may apply a second or higher-order Markov model to enhance the performance of SPAN. Fourth, since we apply the ICM to estimate the model parameters during the model training process, we need to assign each cell/spot to a known cluster type at each iteration. In other words, our model cannot handle unknown cluster types. We will solve this matter in our future studies.

## Supplementary Material

btad641_Supplementary_Data

## Data Availability

The data underlying this article are available in the article and in its online supplementary material.
