# Supplementary material for: Hidden Markov random field models for cell-type assignment of spatially resolved transcriptomics
Source: Bioinformatics. 2023 Nov 7;39(11):btad641. doi: 10.1093/bioinformatics/btad641 (PMC10640398; doi:10.1093/bioinformatics/btad641)
Supplement: btad641_Supplementary_Data [file btad641_supplementary_data.zip › Supplementary.pdf]

Supplementary Figures

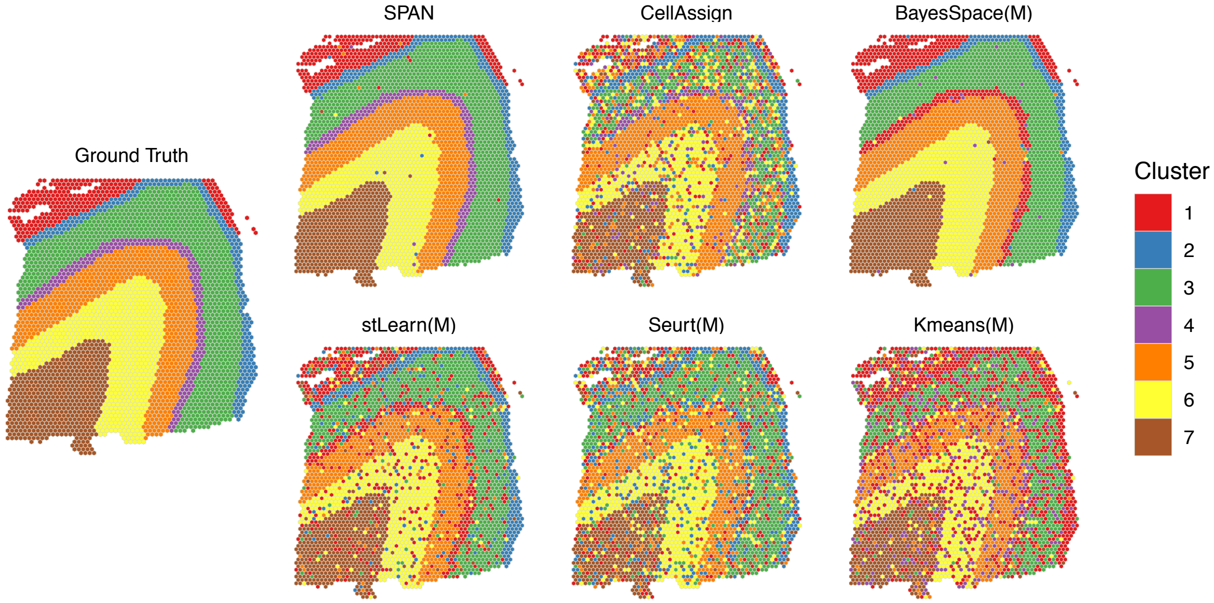

Supplementary Figure S1. Ground truth and predicted cluster assignments generated by different methods on simulated datasets. All methods use marker genes as input.

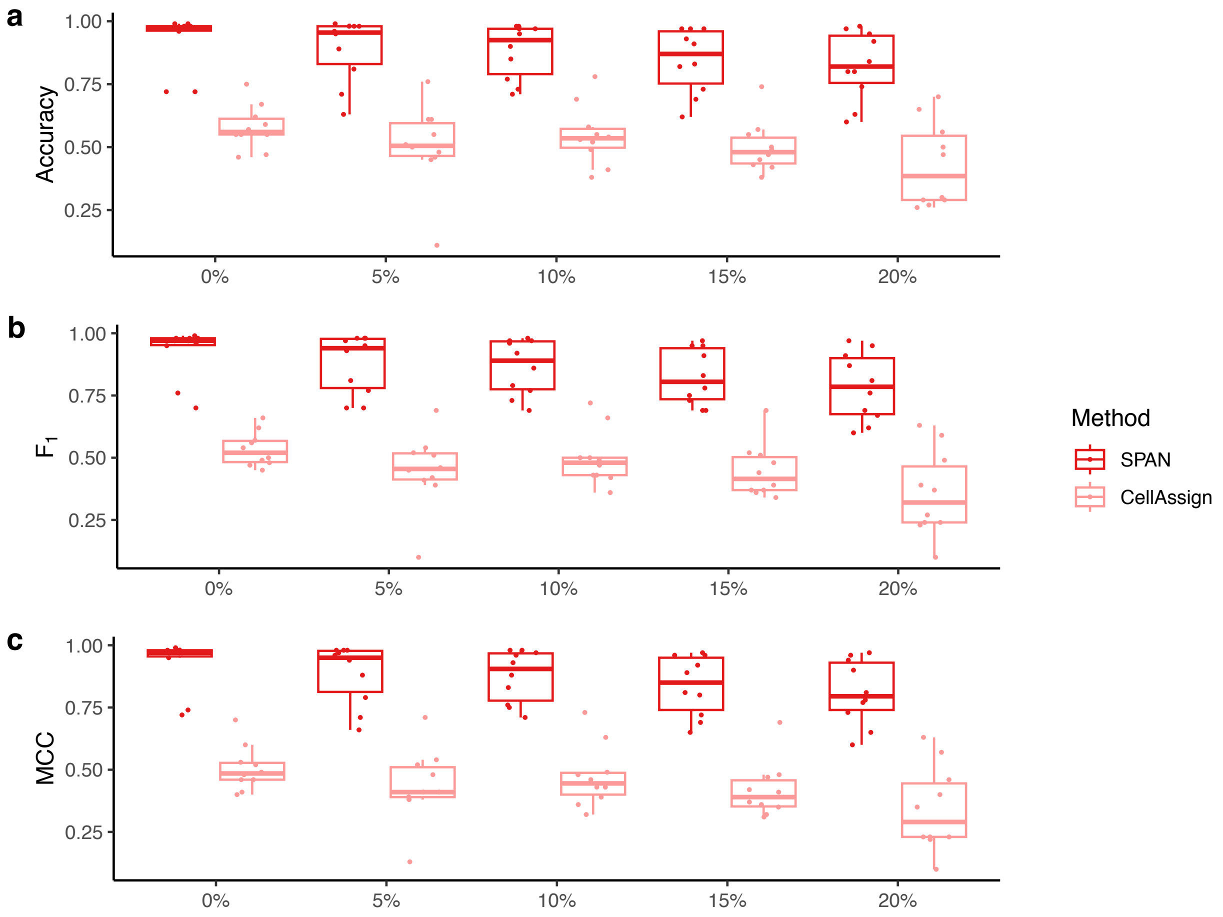

Supplementary Figure S2. Performance on simulated data with various level of inaccurate assigned marker genes. (**a**) Accuracy. (**b**) F1 score. (**c**) MCC.

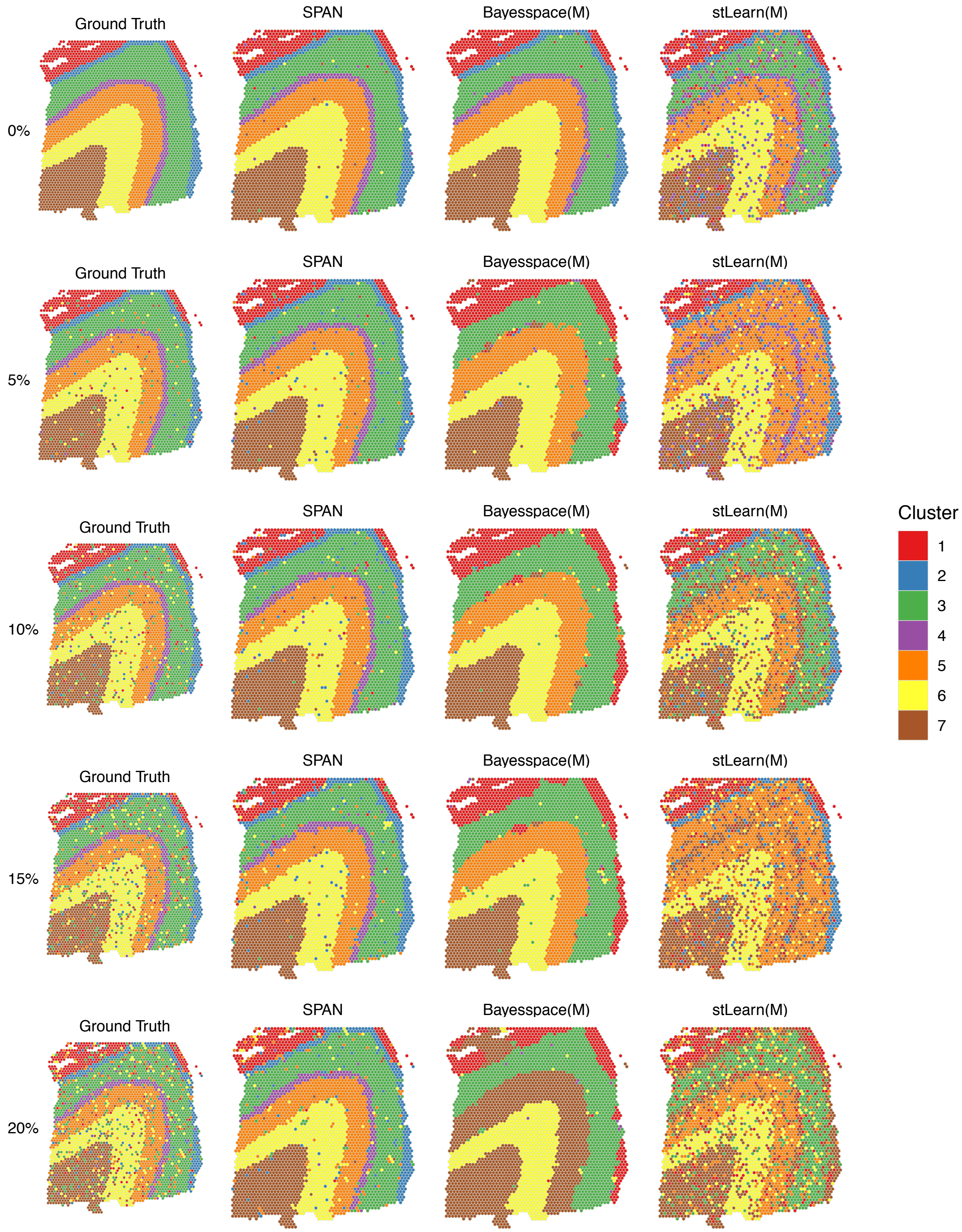

Supplementary Figure S3. Ground truth and predicted cluster assignments generated by different spatial clustering methods on simulated datasets under different levels of spatial noise. All methods use marker genes as input.

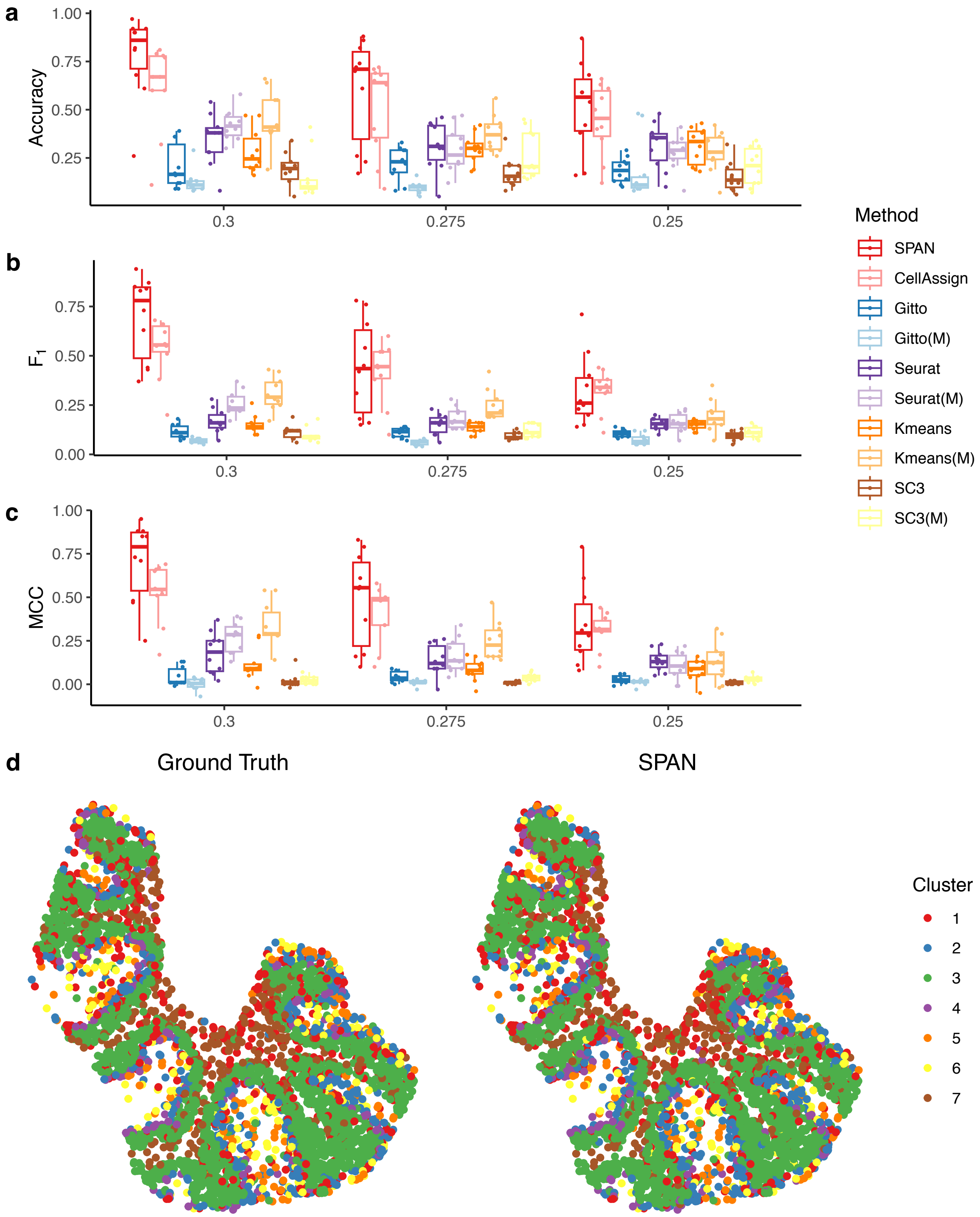

Supplementary Figure S4. Performance on simulated data with various signal strength, where some cell types are mixed. (**a**) Accuracy. (**b**) F1 score. (**c**) MCC. (**d**) Ground truth and predicted cluster assignment generated by SPAN.

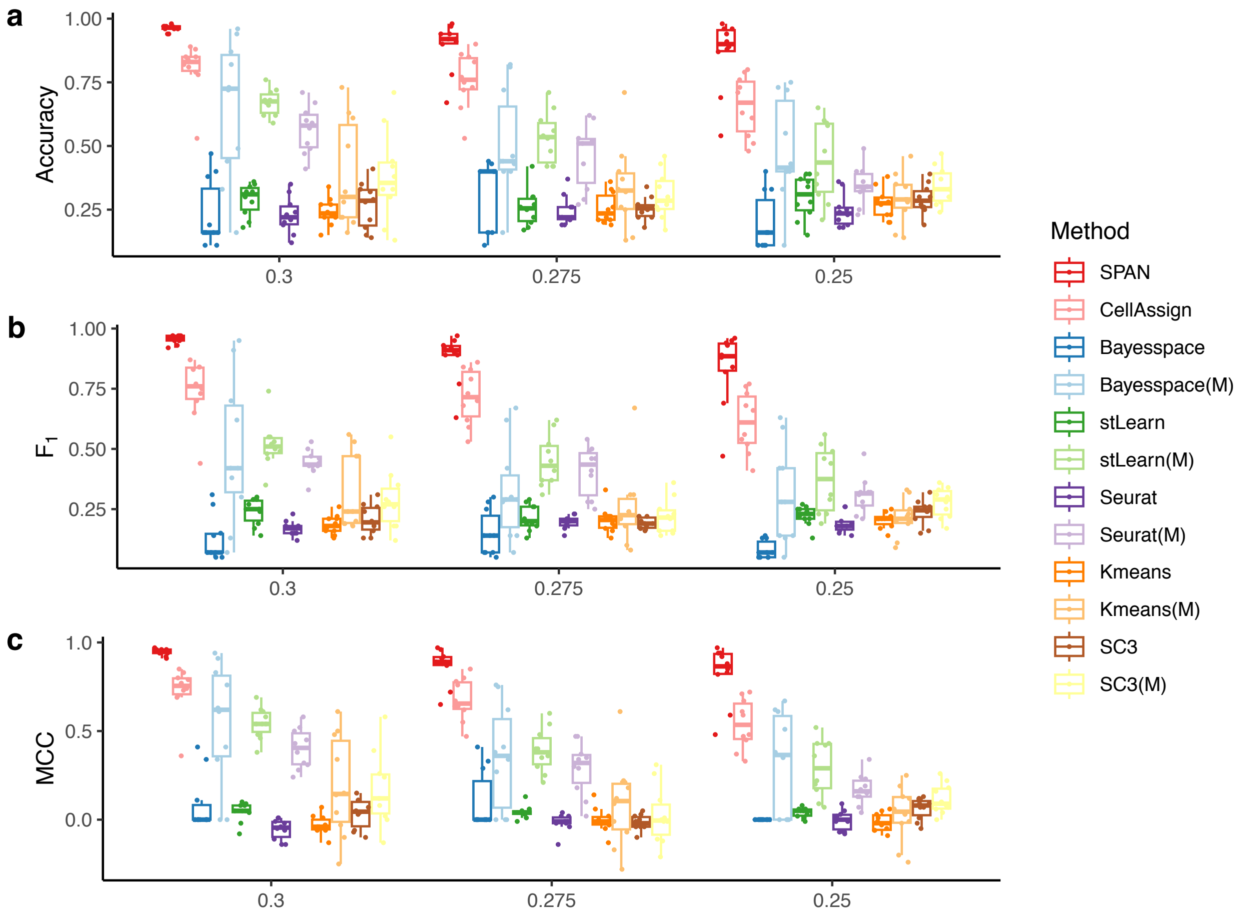

Supplementary Figure S5. Performance on simulated data with various signal strength. The spatial information is extracted from the melanoma dataset (Thrane et al., 2018).
(**a**) Accuracy. (**b**) F1 score. (**c**) MCC.

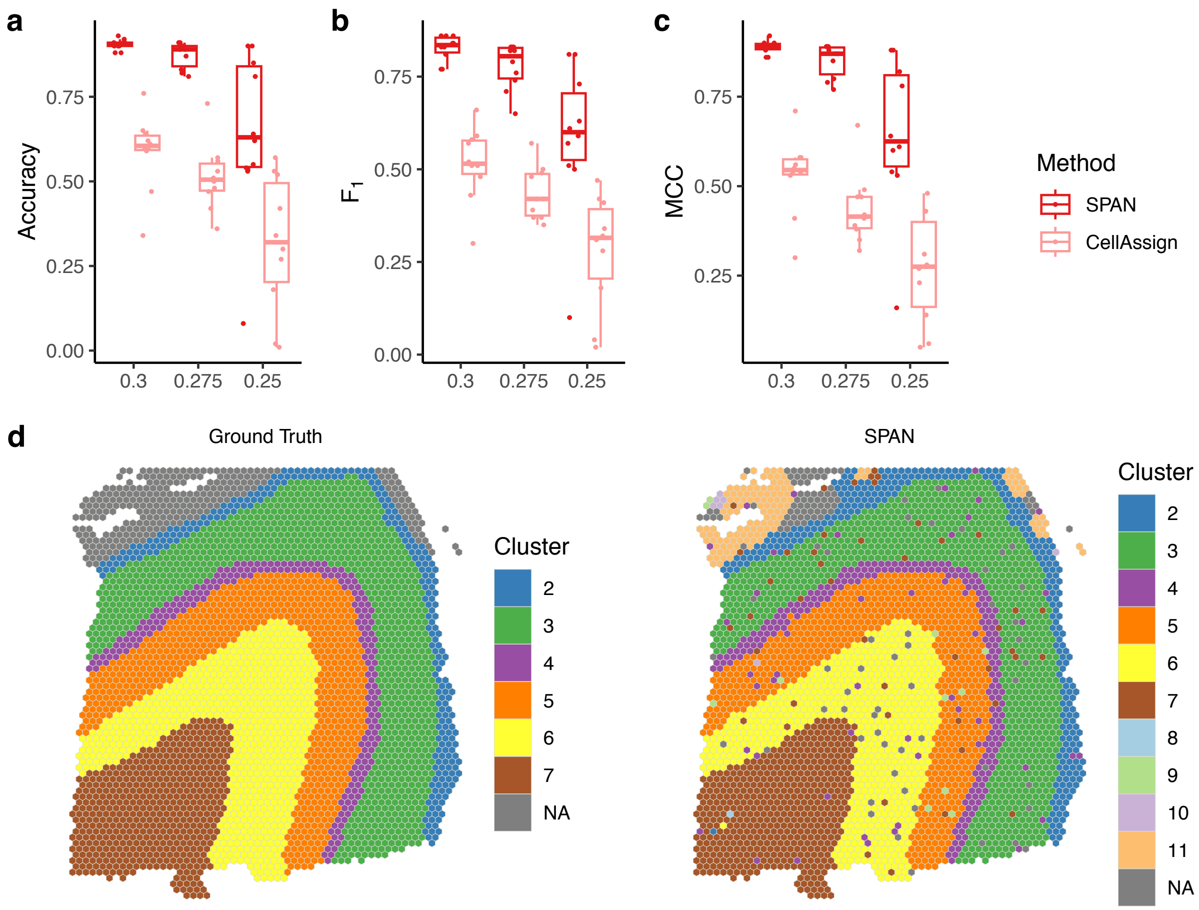

Supplementary Figure S6. Performance on simulated data with various signal strength, where one cell type marker gene information is missing, and four additional non-existent cell types are included. (**a**) Accuracy. (**b**) F1 score. (**c**) MCC. (**d**) Ground truth and predicted cell type assignment generated by SPAN.

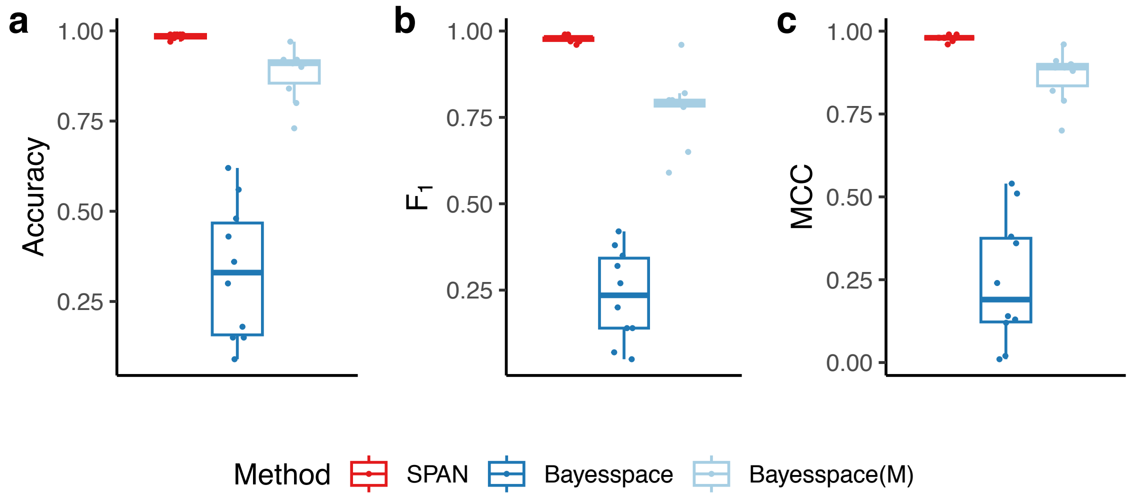

Supplementary Figure S7. Performance on simulated data for batch correction. SPAN uses marker genes as input, while Bayesspace uses HVGs or marker genes (labeled with the letter M in parentheses) as input. (**a**) Accuracy. (**b**) F1 score. (**c**) MCC.

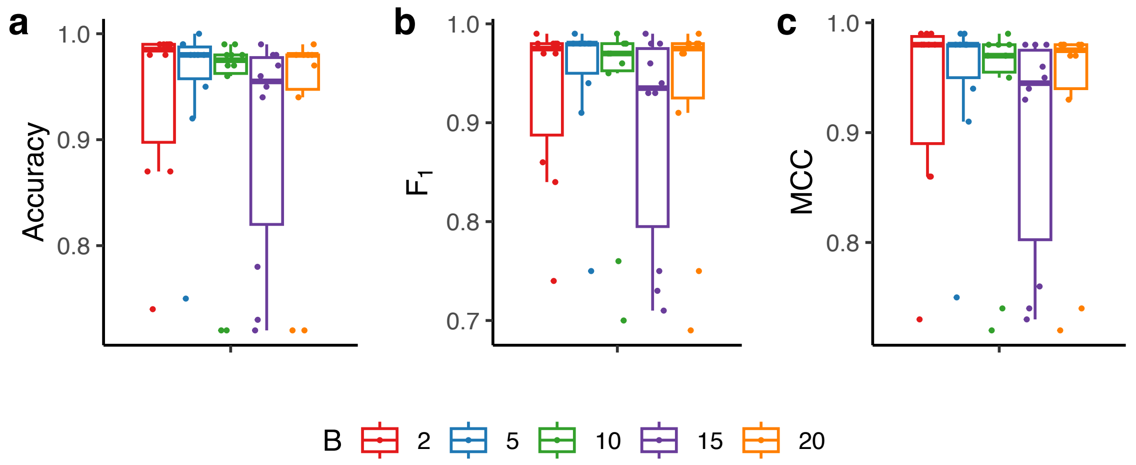

Supplementary Figure S8. Performance on simulated data with different choices of hyperparameter B, for modeling the dispersion of the negative binomial distribution. (**a**) Accuracy. (**b**) F1 score. (**c**) MCC.

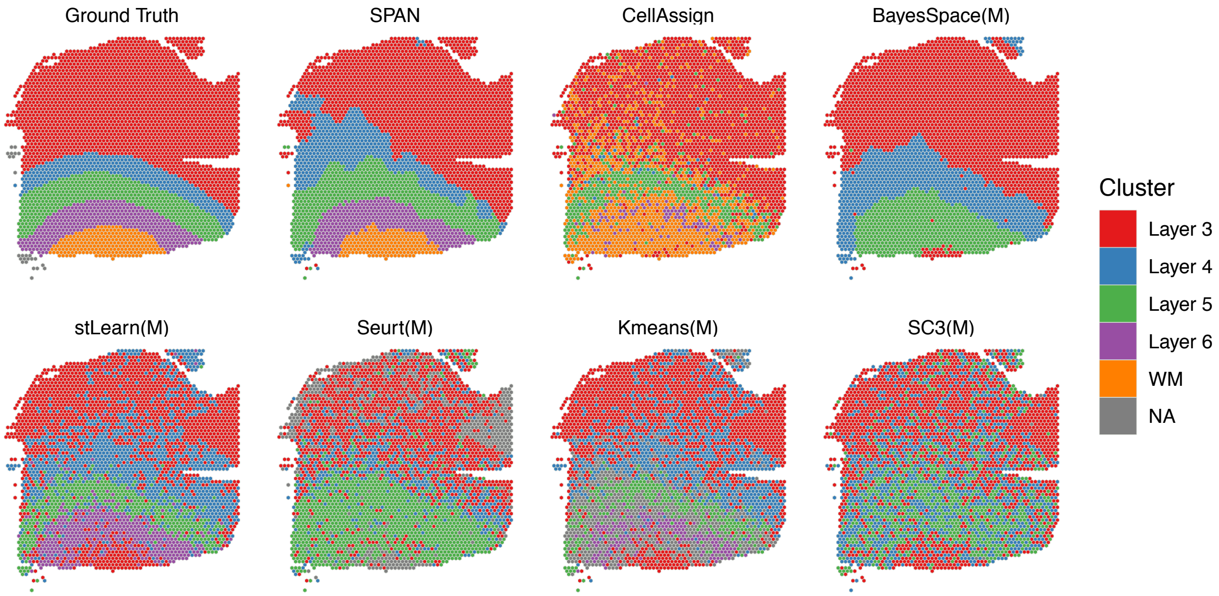

Supplementary Figure S9. Ground truth and predicted cluster assignments generated by different methods of sample 151569 in DLPFC dataset.

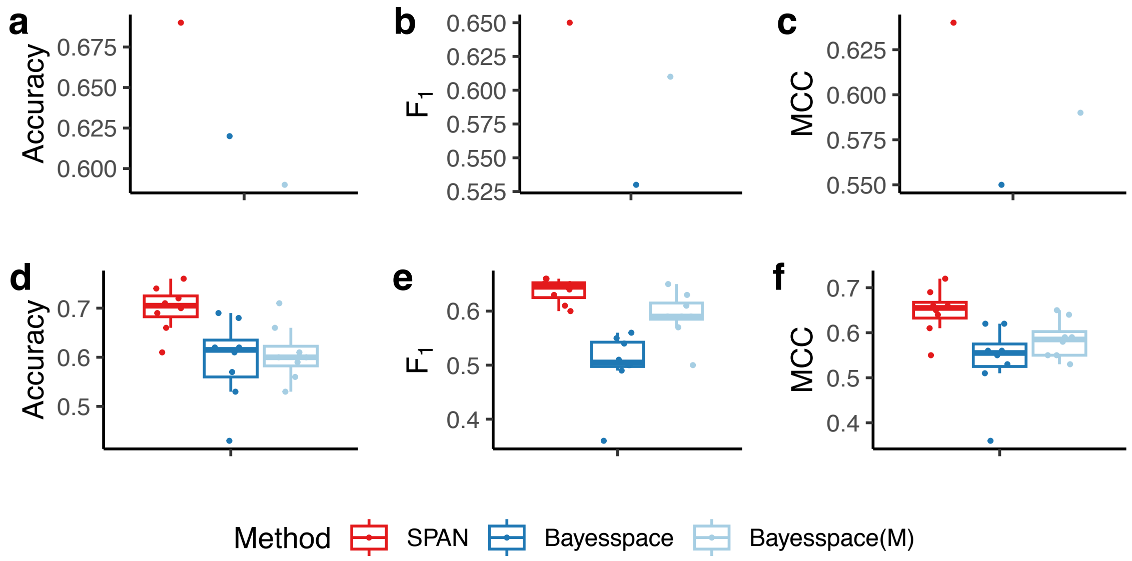

Supplementary Figure S10. Performance for batch correction on DLPFC dataset. SPAN uses marker genes as input, while Bayesspace uses HVGs or marker genes (labeled with the letter M in parentheses) as input. (**a**)(**b**)(**c**) overall performance for the eight batches (samples). (**d**)(**e**)(**f**) performances for each batch (samples) individually.

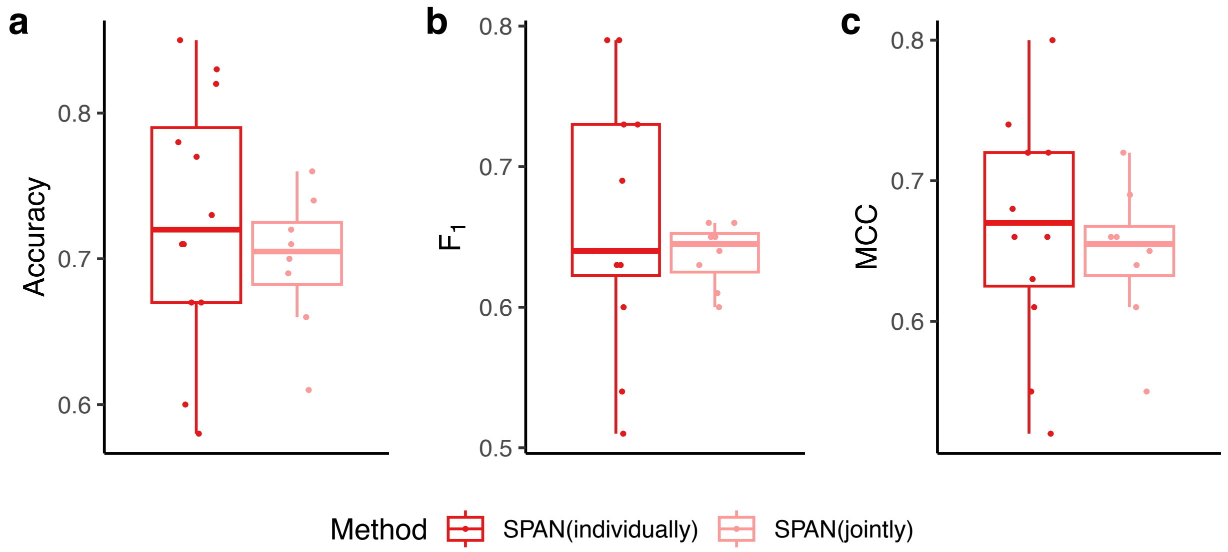

Supplementary Figure S11. Comparison of individually training and jointly training (with batch information as input) on the eight samples of DLPFC dataset. (**a**) Accuracy. (**b**) F1 score. (**c**) MCC.

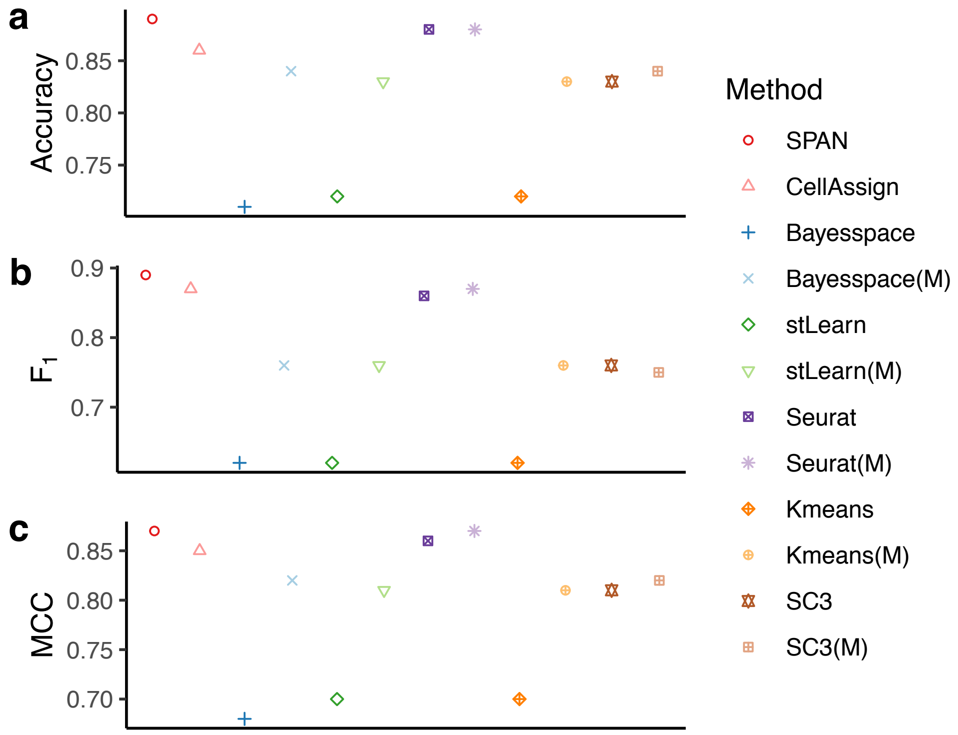

Supplementary Figure S12. Performance on Adult Mouse Brain (FFPE) dataset. SPAN and CellAssign use marker genes as input, while other competing methods use HVGs or marker genes (labeled with the letter M in parentheses) as input. (**a**) Accuracy. (**b**) F1 score. (**c**) MCC.

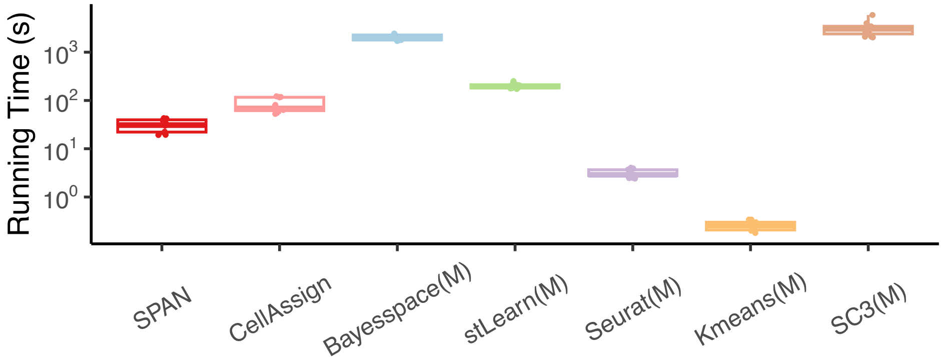

Supplementary Figure S13. Comparison of algorithm running time for 12 samples on DLPFC dataset. All methods use marker genes as input. The experiments are conducted on NVIDIA Tesla P100 GPU and Intel Xeon E5-2630 v4 CPU.

Supplementary Note 1. Detailed description of the simulation settings

To evaluate the model performance that incorporates marker genes and spatial information to identify cell types, we employed a two-step approach to simulate spatial data. We first simulate the scRNA-seq data using R package Splatter (Zappia et al., 2017) and subsequently map each simulated cell to the corresponding spatial location. The details of the parameters used for simulating different scenarios of scRNA-seq data are listed below. We repeated all experiments ten times with different random seeds under the same setting. Moreover, the number of cells per group and the spatial locations or coordinates of cells are derived from real datasets. Specifically, we initially determine the number of cells of different types based on the derived spatial information. Then, we simulate scRNA-seq data with the same number of groups. These cells are then randomly assigned to the locations corresponding to the groups. In the simulation, we extract spatial information from three real datasets, sample 151673 and 151674 in the DLPFC (Maynard et al., 2021), melanoma dataset (Thrane et al., 2018), and Slide-seq cerebellum dataset from RCTD (Cable et al., 2022). Neighbor relations can be directly inferred from the DLPFC dataset and melanoma dataset, while we use the k-nearest neighbor method to generate the neighbor relations from the Slide-seq cerebellum dataset.

To simulate the spatial gene expression, we extract the spatial information from the sample 151673 in the DLPFC (Maynard et al., 2021) dataset. We use Splatter to then generate the raw count matrix of 3611 spots of 2500 genes. The number of spots of each simulated cluster is the same as the number of spots of seven cluster types in the 151673 sample in the DLPFC. The parameters for Splatter are set as dropout.shape = -1, dropout.mid = 2, de.prob = 0.05 and de.facScale = (0.3, 0.275, 0.25, 0.225) for different sigma. The other parameters are set to default values.

To simulate the effect introduced by the noise in the spatial data, we randomly switch the gene expressions and related spot type between different groups. We fix the parameters of Splatter as de.facScale=0.275, others remained the same as previously described, and the number of the randomly switched spots are set as (0.05, 0.1, 0.15, 0.2) times the total number of spots.

To evaluate the performance under a different number of non-marker genes, we replace a portion of marker genes with other randomly selected non-marker genes and randomly assign a group type to each fake marker. We set the parameters of Splatter as de.facScale=0.275, others remained the same. The number of the randomly assigned marker genes are set as (0.05, 0.1, 0.15, 0.2) times the total number of marker genes.

To evaluate the performance under a different number of marker genes assigned to incorrect markers, we replace a portion of marker genes with other randomly selected non-marker genes and randomly assign a group type to each fake marker. We set the parameters of Splatter as

de.facScale=0.275, others remained the same. The number of the randomly assigned marker genes are set as (0.05, 0.1, 0.15, 0.2) times the total number of marker genes.

For the simulation experiments of batch effect, we generate 3611 and 3635 spots from different two batches and extract the spatial information from the sample 151673 and 151674 in the DLPFC datasets for these two batches, respectively. We set the parameters of Splatter as de.facScale=0.275, batch.facLoc = 0.2, and others remained same as previously described. To extract the marker gene indicator matrix $\boldsymbol{\rho}$, we select the marker gene g for each group k if their $DEFacGroup_{gk} > 1.5$.

Simulate absence of one cell type marker gene information and the inclusion of additional four non-existent cell types

To simulate misspecified inputs that can reflect real-world scenarios, we conducted experiments on simulated datasets with the absence of one cell type marker gene information and the inclusion of additional four non-existent cell types. To simulate a scenario where a marker gene is not provided, we denote the Layer 1 as the cell type whose marker gene information is not provided and modify the marker-cell type indicator matrix ρ such that the corresponding column for Layer 1 is set to 0. This indicates that none of marker genes are overexpressed in the Layer 1. To simulate the non-existent cell types in the dataset, we assume that the marker genes corresponding to such cell types are not overexpressed in the existing cell types. Subsequently, we generate 100 non-informative genes, evenly distributed across four additional groups, combine them with the marker genes associated with the existing cell types and make corresponding adjustments to the marker-cell type indicator matrix ρ. These are utilized as the input for SPAN and Cellassign.

Simulate data where different cell types are mixed

To simulate the data where different cell types are mixed, we extract the spatial information from Slide-seq cerebellum dataset from RCTD (Cable et al., 2022). We randomly sample 40% of cells from the seven most common cell types of the dataset to obtain the coordinates of 4122 cells from seven distinct cell types. We constructed the neighbor structure by finding the k-nearest neighbors of each cell, with k=15, to derive the cell neighbor relationship.

Supplementary Note 2. Detailed description of the real SPT data

The raw count and spatial information of the DLPFC dataset (Maynard et al., 2021) were downloaded from R package Bayesspace (Zhao et al., 2021). The authors analyzed the data and identified the cell types. The dataset has 12 samples, each of which has 3000 to 4000 spots with 33538 genes from 5 to 7 groups.

The Adult Mouse Brain (FFPE) datasets were downloaded from the 10X genomics (Zheng et al., 2017) website (https://www.10xgenomics.com/resources/datasets/adult-mouse-brain-ffpe-1-standard-1-3-0). We use the graph-based clustering results obtained from the analysis webpage as the clustering ground truth for the Adult Mouse Brain dataset. The dataset has 2264 spots with 19465 genes from 9 groups.

The osmFISH (mouse cortex) dataset (Codeluppi et al., 2018) was downloaded from the website (http://linnarssonlab.org/osmFISH/). This dataset contains 4839 cells with 33 genes from 11 groups. We did not perform the feature selection for this dataset due to the low dimension of features.

The spatial information of the melanoma dataset (Thrane et al., 2018) was derived from R package Bayesspace (Zhao et al., 2021). The authors analyzed the data and identified the cell types. The dataset has 293 spots from 4 groups.

The spatial information Slide-seq cerebellum dataset from RCTD (Cable et al., 2022) was derived from the website (https://github.com/dmcable/spacexr/tree/master). We randomly sample 40% of cells from the seven most common cell types (Astrocytes, Bergmann, Granule, Purkinje, MLI2, MLI1 and Oligodendrocytes) of the dataset to obtain the coordinates of 4122 cells from seven distinct cell types.

To extract the marker genes, we implemented the differential expression analysis using the DESeq from the R package DESeq2 (Love et al., 2014). For each cluster, we sort the log fold change value of genes with adjusted p-value less than 0.05 in descending order. The top N genes were selected as the marker genes for the respective cluster. For Adult Mouse Brain dataset, we set N = 30, and obtained 259 marker genes in total. The select marker genes are listed in **Supplementary Table S1**.

Supplementary Note 3. Ablation study on the hyperparameter B

In the experiment, we followed Cellassign (Zhang et al., 2019) to set the default value of hyperparameter B, which models the dispersion of the negative binomial distribution, to 10. We undertook an ablation study to evaluate the model performance under varied values of B. We employ the simulation settings using the spatial information from DLPFC dataset, fix sigma and range B from 2 to 20. As illustrated in **Supplementary Figure S8**, our model can achieve a relatively stable performance under different values of B.

Reference

Cable, D. M., Murray, E., Zou, L. S., Goeva, A., Macosko, E. Z., Chen, F., & Irizarry, R. A. (2022). Robust decomposition of cell type mixtures in spatial transcriptomics. *Nat Biotechnol*, *40*(4), 517-526. https://doi.org/10.1038/s41587-021-00830-w

Codeluppi, S., Borm, L. E., Zeisel, A., La Manno, G., van Lunteren, J. A., Svensson, C. I., & Linnarsson, S. (2018). Spatial organization of the somatosensory cortex revealed by osmFISH. *Nature Methods*, *15*(11), 932-935.

Love, M. I., Huber, W., & Anders, S. (2014). Moderated estimation of fold change and dispersion for RNA-seq data with DESeq2. *Genome biology*, *15*(12), 1-21.

Maynard, K. R., Collado-Torres, L., Weber, L. M., Uytingco, C., Barry, B. K., Williams, S. R., Catallini, J. L., 2nd, Tran, M. N., Besich, Z., Tippani, M., Chew, J., Yin, Y., Kleinman, J. E., Hyde, T. M., Rao, N., Hicks, S. C., Martinowich, K., & Jaffe, A. E. (2021). Transcriptome-scale spatial gene expression in the human dorsolateral prefrontal cortex. *Nat Neurosci*, *24*(3), 425-436. https://doi.org/10.1038/s41593-020-00787-0

Thrane, K., Eriksson, H., Maaskola, J., Hansson, J., & Lundeberg, J. (2018). Spatially Resolved Transcriptomics Enables Dissection of Genetic Heterogeneity in Stage III Cutaneous Malignant Melanoma. *Cancer Res*, *78*(20), 5970-5979. https://doi.org/10.1158/0008-5472.CAN-18-0747

Zappia, L., Phipson, B., & Oshlack, A. (2017). Splatter: simulation of single-cell RNA sequencing data. *Genome Biol*, *18*(1), 174. https://doi.org/10.1186/s13059-017-1305-0

Zhang, A. W., O'Flanagan, C., Chavez, E. A., Lim, J. L. P., Ceglia, N., McPherson, A., Wiens, M., Walters, P., Chan, T., Hewitson, B., Lai, D., Mottok, A., Sarkozy, C., Chong, L., Aoki, T., Wang, X., Weng, A. P., McAlpine, J. N., Aparicio, S., Steidl, C., Campbell, K. R., & Shah, S. P. (2019). Probabilistic cell-type assignment of single-cell RNA-seq for tumor microenvironment profiling. *Nat Methods*, *16*(10), 1007-1015. https://doi.org/10.1038/s41592-019-0529-1

Zhao, E., Stone, M. R., Ren, X., Guenthoer, J., Smythe, K. S., Pulliam, T., Williams, S. R., Uytingco, C. R., Taylor, S. E. B., Nghiem, P., Bielas, J. H., & Gottardo, R. (2021). Spatial transcriptomics at subspot resolution with BayesSpace. *Nat Biotechnol*, *39*(11), 1375-1384. https://doi.org/10.1038/s41587-021-00935-2

Zheng, G. X., Terry, J. M., Belgrader, P., Ryvkin, P., Bent, Z. W., Wilson, R., Ziraldo, S. B., Wheeler, T. D., McDermott, G. P., Zhu, J., & others. (2017). Massively parallel digital transcriptional profiling of single cells. *Nature communications*, *8*(1), 14049.
